# Supplementary material for: Control over the emerging chirality in supramolecular gels and solutions by chiral microvortices in milliseconds
Source: Nat Commun. 2018 Jul 3;9:2599. doi: 10.1038/s41467-018-05017-7 (PMC6030102; doi:10.1038/s41467-018-05017-7)
Supplement: Supplementary file 1 — Supplementary Information [file 41467_2018_5017_MOESM1_ESM.pdf]

Supplementary Information

Control over the Emerging Chirality in Supramolecular Gels and Solutions by Chiral Microvortices in Milliseconds

Sun et al.

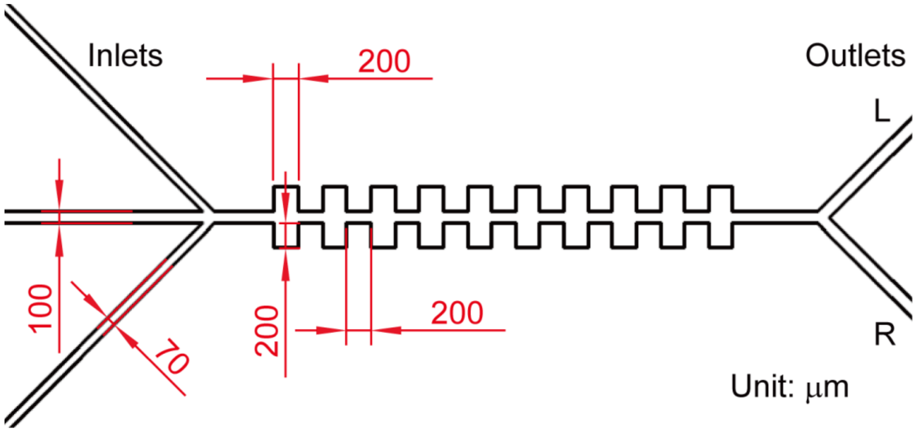

Supplementary Figure 1 | Microchannel design. The microchannel consists of ten pairs of inclined microchambers with three inlets and two outlets. L (R) indicates the left (right) outlet along the flow direction. The height of microchannel is 50 μm.

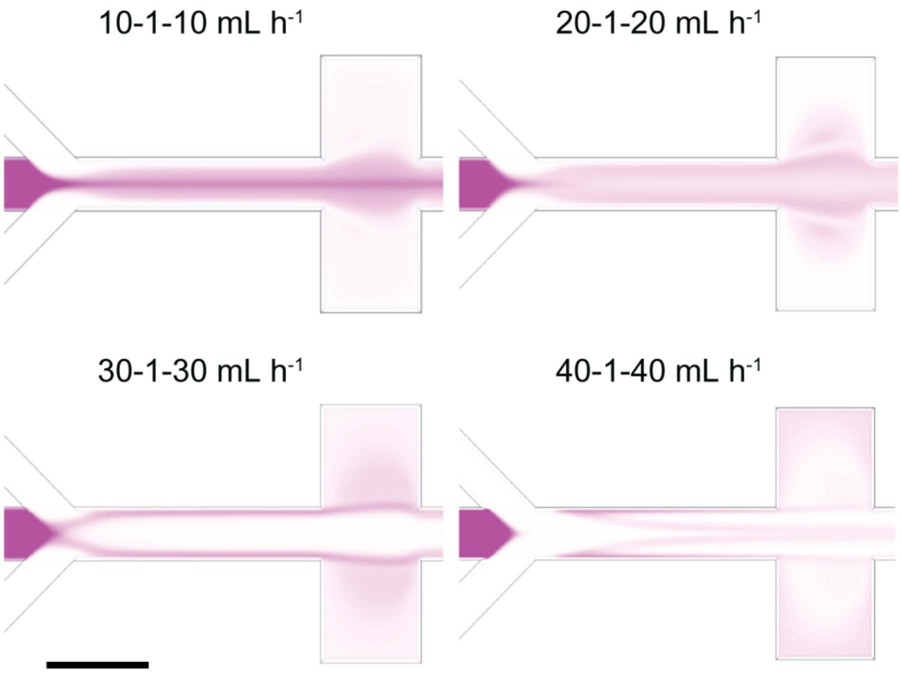

Supplementary Figure 2 | Numerical simulations of fluid motion and species transportation at various flow rates. The simulations are performed at a fixed flow rate of 1 mL h^{-1} for the middle inlet and various flow rates of 10, 20, 30, and 40 mL h^{-1} for each side inlet. The middle infusion is denoted by pink color. Scale bar, 200 μm.

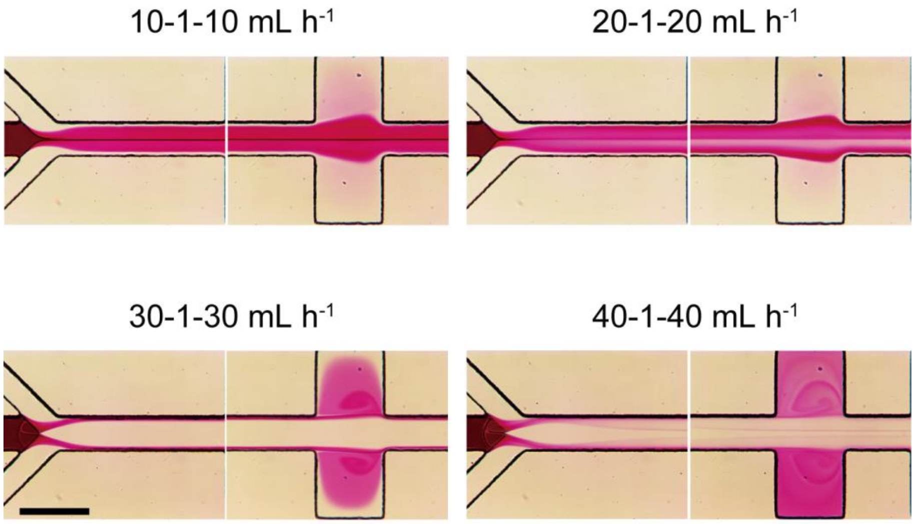

Supplementary Figure 3 | Bright-field images of fluid motion and species transportation at various flow rates. The fluid motion and species transport are visualized by spiking xylenol orange (50 mM, pink color) into the middle infusion. The bright-field observation is recorded at a fixed flow rate of 1 mL h^{-1} for the middle inlet and various flow rates of 10, 20, 30, and 40 mL h^{-1} for each side inlet. Scale bar, 200 μm.

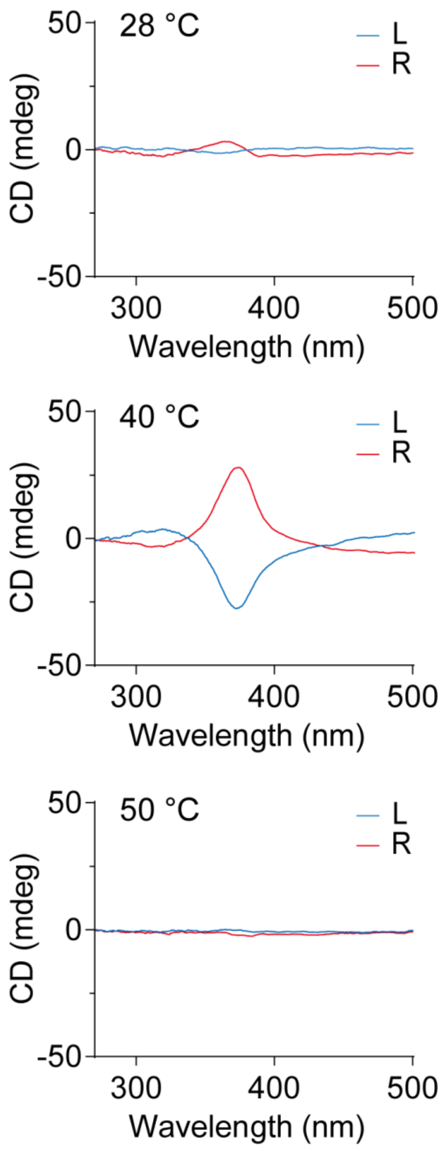

Supplementary Figure 4 | Temperature optimization for the self-assembly of BTAC molecules. Opposite CD signals of BTAC assemblies from the L- (blue line) and R-outlet (red line) are obtained only at 40 ℃. The BTAC concentration of the middle inlet is 61 mg mL^{-1}. The flow rate of each side inlet for DMF/H_2O solution is 30 mL h^{-1} and that of the middle inlet for the BTAC solution is 1 mL h^{-1}.

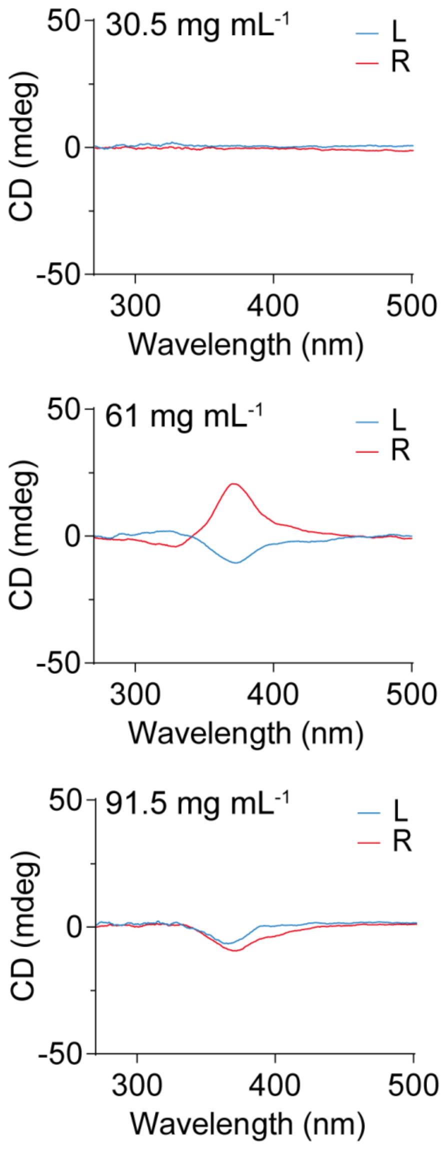

Supplementary Figure 5 | Optimization of BTAC concentration. Opposite CD signals of BTAC assemblies from the L- (blue line) and R-outlet (red line) are obtained only at the BTAC concentration of 61 mg mL^{-1}. The temperature of water bath is maintained at 40 ℃. The flow rate of each side inlet for DMF/H_2O solution is 30 mL h^{-1} and that of the middle inlet for the BTAC solution is 1 mL h^{-1}.

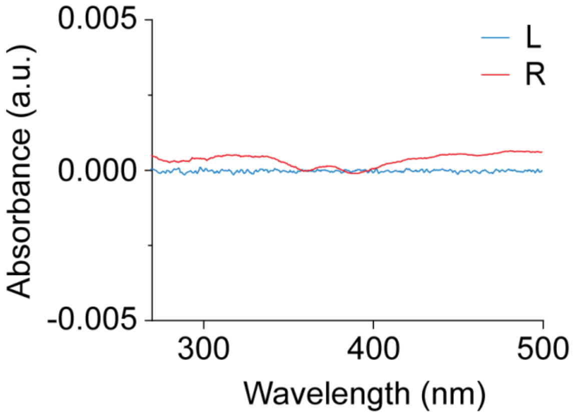

Supplementary Figure 6 | No LD signals for the BTAC assemblies. Blue (red) line refers to measurement from the gels collected from the L- (R-) outlet.

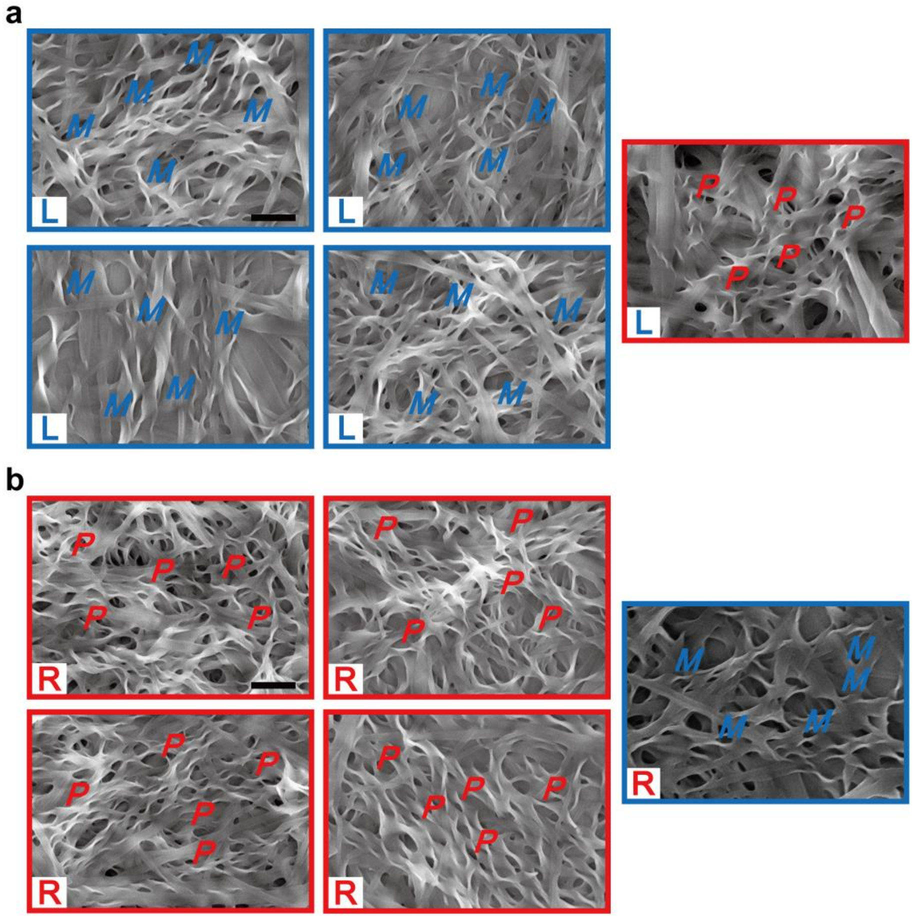

Supplementary Figure 7 | SEM images of BTAC gels. Predominant amounts of **a,** left- (*M*, 80 %) and **b,** right-handed twists (*P*, 86 %) are observed in BTAC gels from the L- and R-outlet. Scale bars, 1 μm.

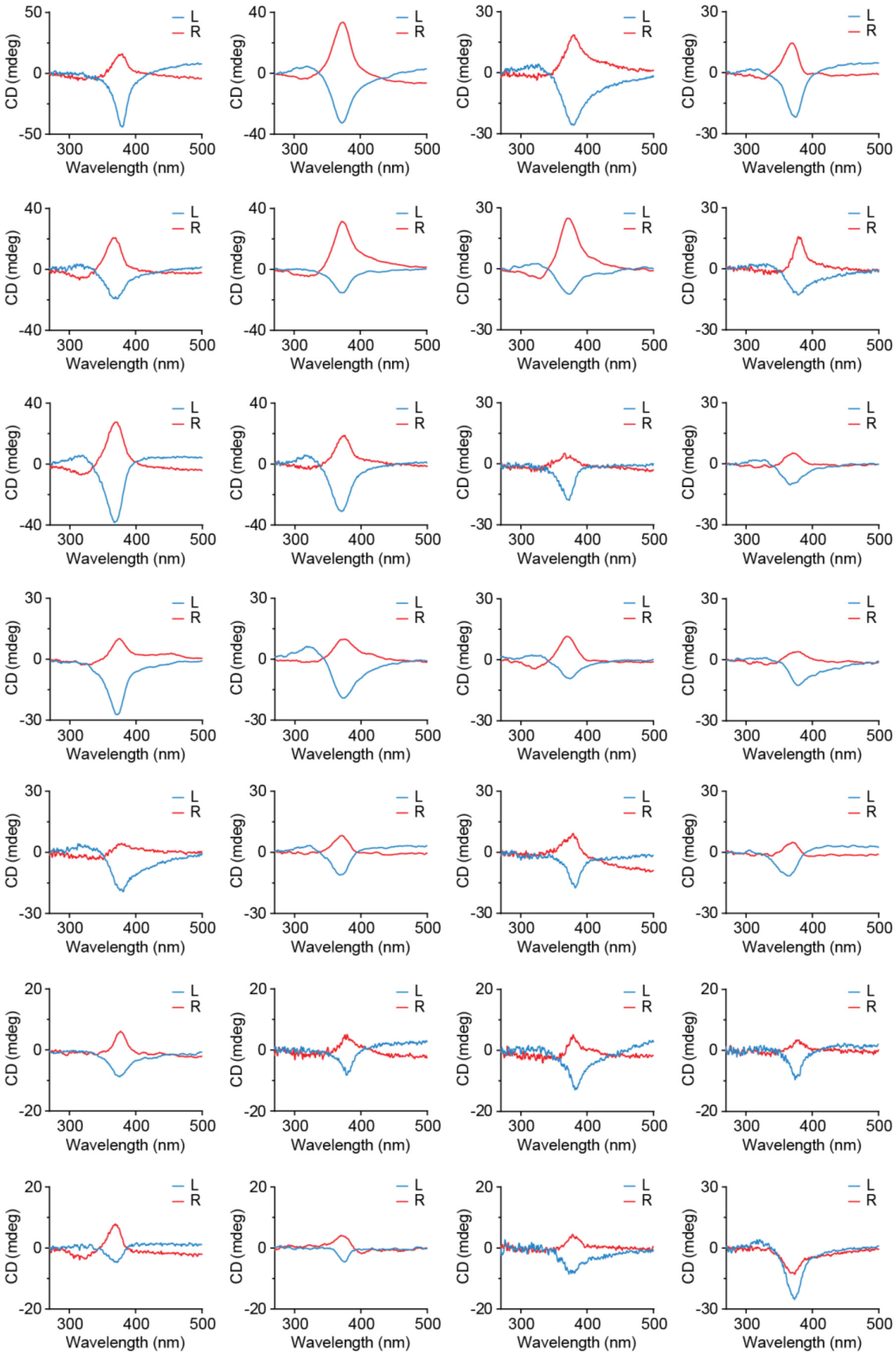

Supplementary Figure 8 | Almost absolute chirality control of BTAC gels. The CD spectra of 28 independent experiments display 28 negative and 27 positive CD signals from the L- (blue lines) and R-outlet (red lines).

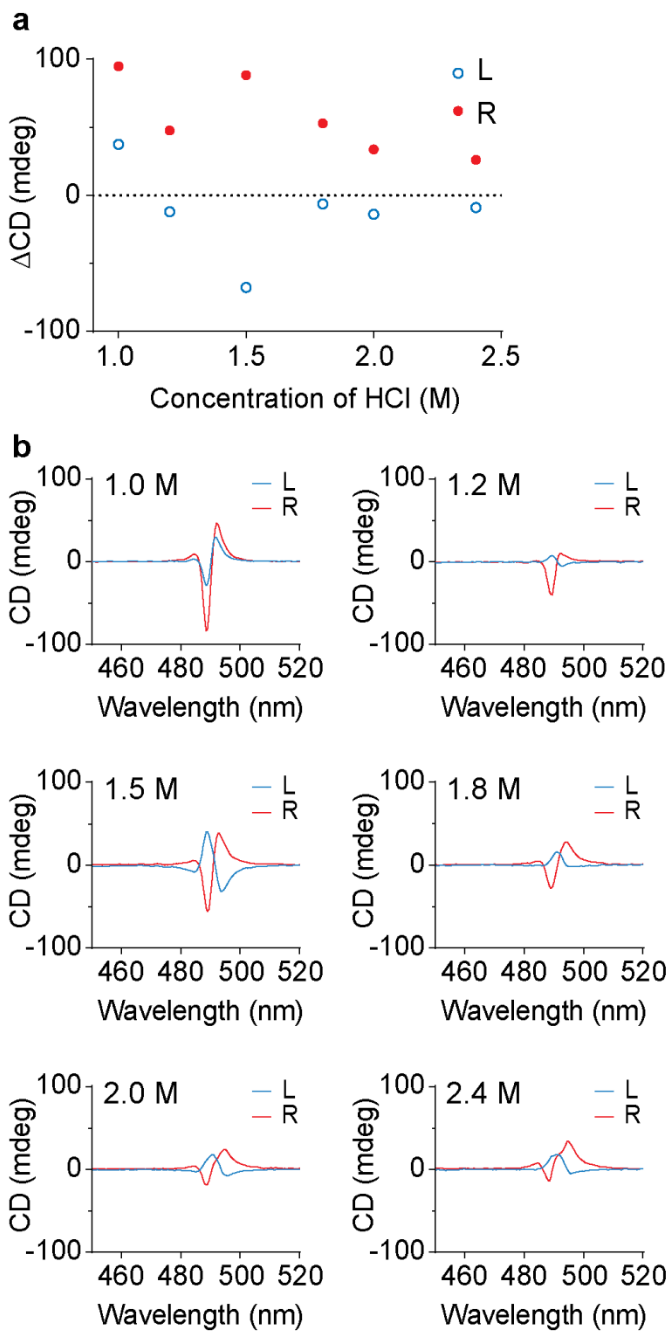

Supplementary Figure 9 | Optimization of HCl concentration for the self-assembly of $TPPS_4$ molecules. a, CD signals and **b,** CD spectra of the $TPPS_4$ aggregates collected from the L- (blue lines) and R-outlet (red lines) at various HCl concentrations ranging from 1.0 to 2.4 M. The flow rate of each side inlet for $TPPS_4$ solution (20 μM) is 30 mL h^{-1} and that of the middle inlet for the mixture solution (HCl + 0.4 M C_2mim^+) is 1 mL h^{-1}.

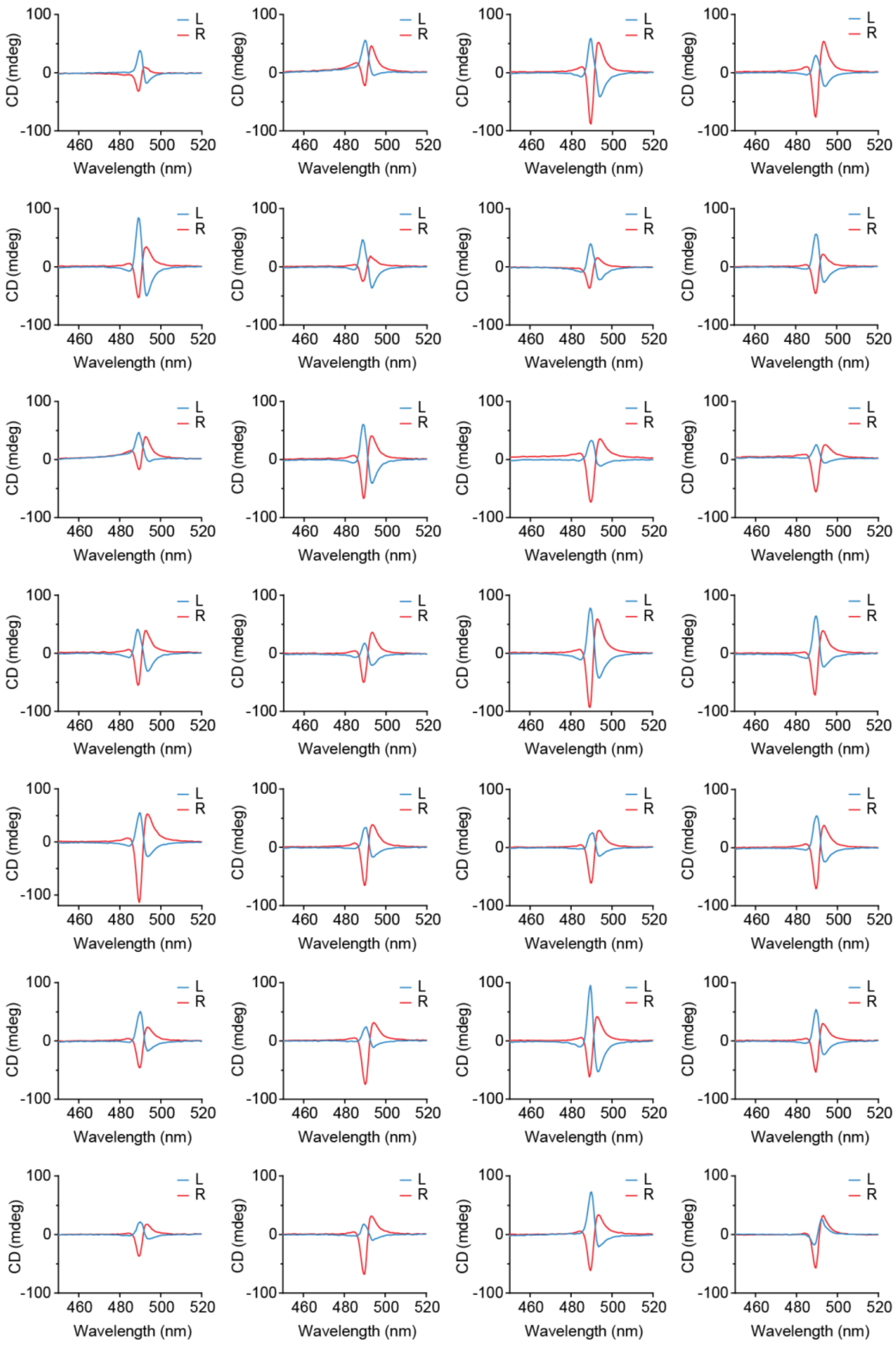

Supplementary Figure 10 | Almost absolute chirality control of $TPPS_4$ assemblies.

The CD spectra of 28 independent experiments display 27 negative and 28 positive CD signals of $TPPS_4$ assemblies from the L- (blue lines) and R-outlet (red lines).

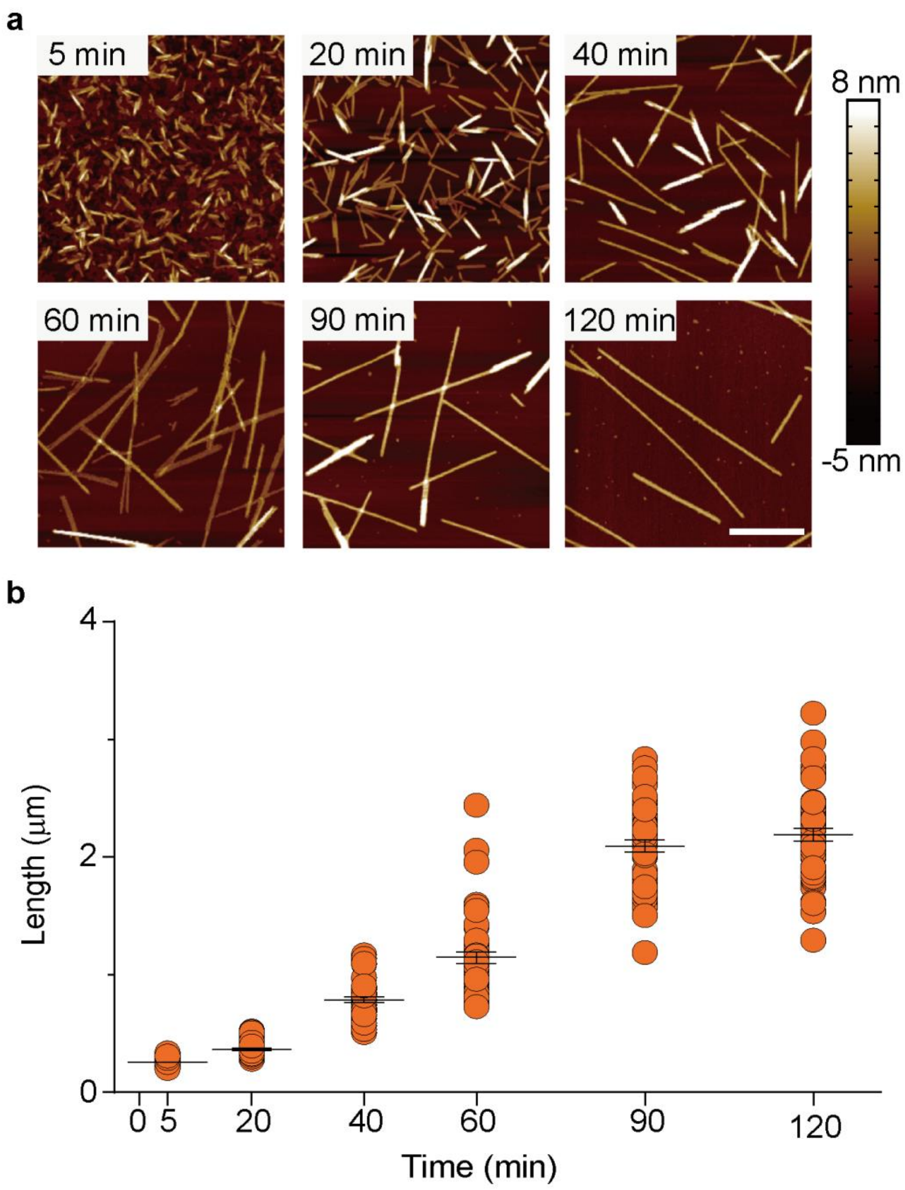

Supplementary Figure 11 | Assembly kinetics of $TPPS_4$ aggregates. a, AFM characterization of $TPPS_4$ aggregates at different time intervals. Scale bar, 1 μm. **b,** Time-dependent length growth (orange dots) of $TPPS_4$ nanotubes (n = 50 for each time point, mean ± s.e.m.).

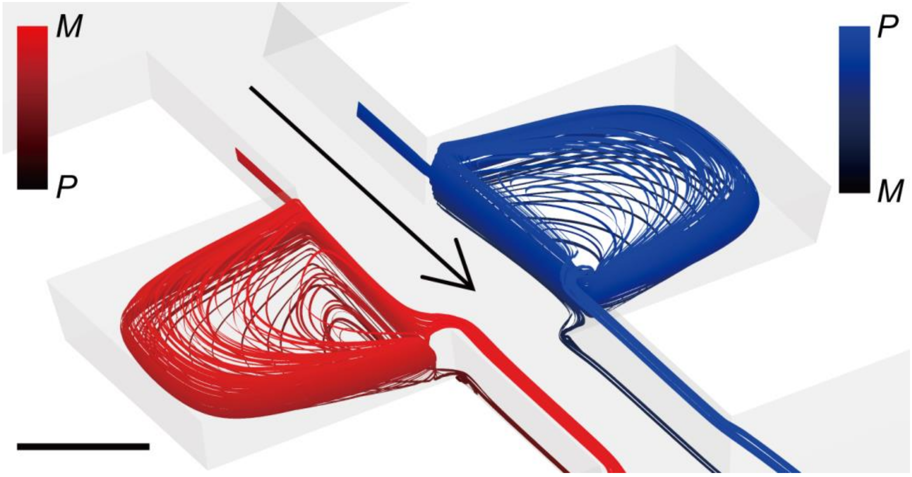

Supplementary Figure 12 | Simulation results of microvortices with opposite chirality signs. Majority of microvortices tend to spiral upward, determining a predominantly *P* (*M*) chirality sign for microvortices in the left (right) chamber. Scale bar, 100 μm.

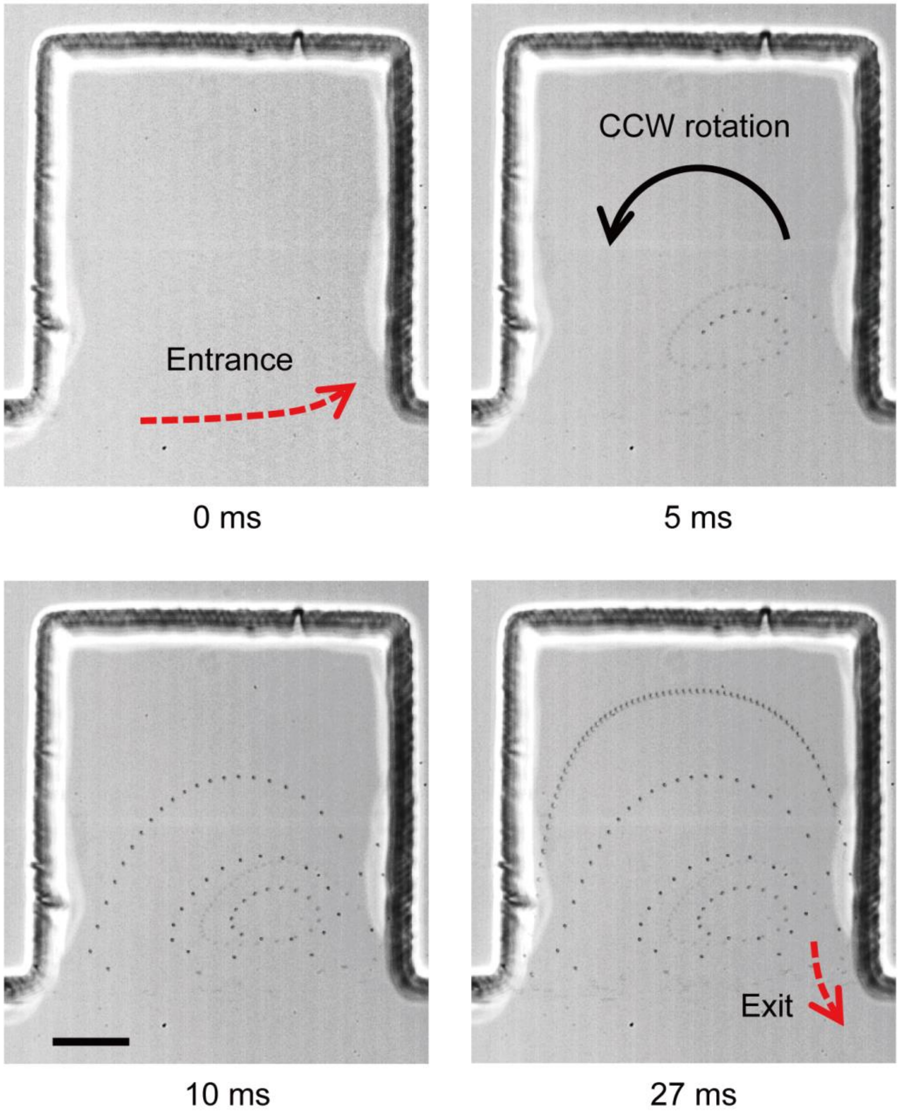

Supplementary Figure 13 | Trajectory of a single tracer particle in the inclined microchamber. The superimposed trajectory of a tracer particle (1 μm) suggests a short residence time of ~ 27 ms in the microchamber and a high rotation speed of 4×10^4 rpm near the vortex center. Scale bar, 40 μm.

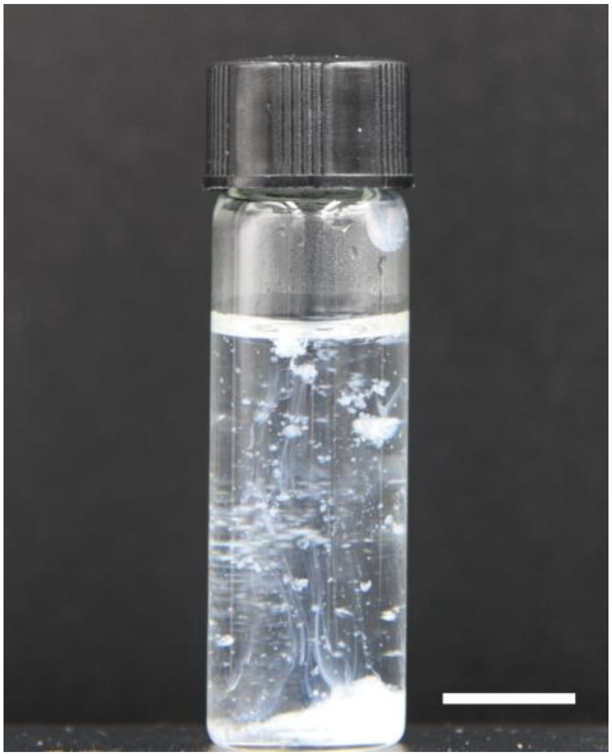

Supplementary Figure 14 | Photograph of BTAC gels collected from the microfluidic outlet. Scale bar, 1 cm.

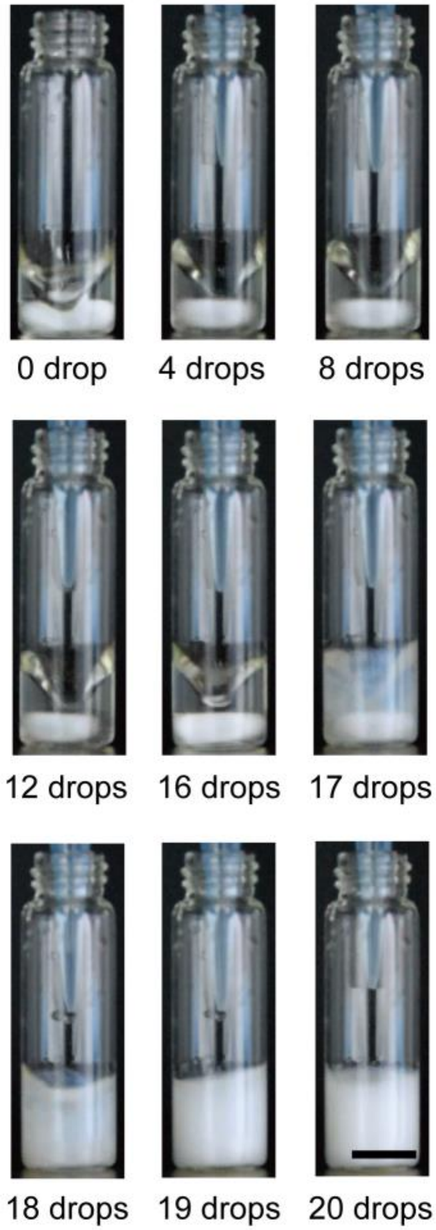

Supplementary Figure 15 | Formation of BTAC gels in stirring cuvettes. Time-series photographs show the gelation process of BTAC by adding H_2O into DMF under CW stirring at 900 rpm. BTAC gels rapidly form when the volume ratio of H_2O to DMF is near 2:5. The average volume of water drops is 20 μL and the DMF volume is 1000 μL. Scale bar, 1 cm.

Supplementary Methods

Master mold fabrication The master molds for the inclined microchannels were manufactured using T3050 photoresist (Baisiyou, China) by MiTASChip (China). A 4 inch silicon wafer was baked at 150 °C for 5 min and left still for 5 min. The T3050 photoresist was spin-coated on the 4 inch wafer at 800 rpm for 35 sec under a vacuumed condition to obtain the desired thickness of ~50 μm. The coated wafer was left still for 2 min to even the surface. The photoresist was exposed to ultraviolet (UV) light for 15 sec through a photomask containing the microchannel patterns (Supplementary Figure 1). The gap between photomask and photoresist was ~80 μm during the exposure. Before and after the exposure, the wafer was baked at: 60 °C for 2 min, 95 °C for 10 min, and 60 °C for 2 min. The wafer was then soaked in a SU-8 developer (MicroChem, USA) to wash away the unexposed photoresist and finally baked at 150 °C for 20 min.

Microchannel fabrication The degassed mixture of PDMS and curing agent (10:1) was cast over the mold and then solidified at 80 °C for 20 min in an oven. After peeling off the PDMS slab from the silicon mold, the inlet and outlet ports were punched through the PDMS using a sharpened 19G dispensing needle (ID 0.75 mm, OD 1 mm). The PDMS was then treated with plasma (at ~55 Pa for 40 sec) and bonded to a glass substrate. Plastic tubes were inserted into the inlet and outlet ports and glued with the adhesive sealant (Dow Corning 3145 RTV, USA).

Optimization of temperature and BTAC concentration For temperature optimization, the microfluidic device was immersed into a water bath at different temperatures of 28 ℃, 40 ℃, and 50 ℃ during the self-assembly process (Supplementary Figure 4). The BTAC concentration in the middle infusion was kept at 61 mg mL^{-1}, leading to an average

concentration of ~ 1 mg mL^{-1} at the outlets under the flow conditions of 30–1–30 mL h^{-1}. The CD spectra showed a negative Cotton effect for the L-outlet and a positive Cotton effect for the R-outlet only at 40 ℃, whereas no opposite CD signals for the opposing outlets were detected at 28 ℃ and 50 ℃.

For optimization of BTAC concentration, two additional BTAC concentrations, 30.5 and 91.5 mg mL^{-1}, were used while keeping the temperature at 40 ℃ (Supplementary Figure 5). No gels were formed and no CD signals were detected for 30.5 mg mL^{-1} BTAC. Using 91.5 mg mL^{-1} BTAC, gels were obtained, but no opposite CD signals were observed for the opposing outlets. Therefore, the BTAC concentration of 61 mg mL^{-1} and water batch temperature of 40 ℃ were used for microfluidic self-assembly of BTAC gels.

Optimization of HCl concentration HCl concentrations ranging from 1.0 M to 2.4 M were used for self-assembly of $TPPS_4$ aggregates within the microfluidic device, while keeping $TPPS_4$ concentration at 20 μM and C_2mim^+ concentration at 0.4 M (Supplementary Figure 9). The CD spectra showed no opposite CD signals for the opposing outlets using 1.0 M HCl. With the increased HCl concentration, opposite CD signals were observed and reached their maximum at 1.5 M HCl. We thus used 1.5 M HCl + 0.4 M C_2mimBF_4 as the mixture solution for the middle infusion in the present study.

Spectra measurement The CD and UV-Vis spectra of BTAC assemblies (270 nm to 500 nm) and $TPPS_4$ assemblies (400 nm to 520 nm) were measured using a JASCO J-1500 spectrometer (Japan). BTAC measurements were performed in a 0.1 mm cuvette using integrations of 1 s, data pitch of 0.5 nm, scanning speed of 500 nm min^{-1}, and single

acquisition with a bandwidth of 5 nm. DMF/H_2O (v/v, 5/2) was used for the baseline correction. $TPPS_4$ measurements were performed in a quartz cuvette with a path length of 3 mm using integrations of 0.5 s, data pitch of 0.5 nm, scanning speed of 200 nm min^{-1}, and single acquisition with a bandwidth of 2 nm. A 1:61 dilution of the initial mixture solution (0.4 M C_2mimBF_4 + 1.5 M HCl) was used for base line correction. The sign and magnitude of CD signal for $TPPS_4$ assemblies were determined as $CD_{493.5nm} - CD_{489.5nm}$.

Scanning electron microscopy (SEM) The SEM characterizations of microchannel structure and BTAC gels were performed on a Hitachi S-4800 FE-SEM (Japan) with an accelerating voltage of 5 kV. Before SEM measurement, the samples on silicon wafers were dried in a vacuumed condition and then coated with a 5 nm Au layer for 30 s to increase the contrast.

Atomic force microscopy (AFM) Fresh mica substrate was pretreated using 100 mM $NiCl_2$ solution for 5 min to facilitate the deposition of the $TPPS_4$ assemblies. A drop of solution containing $TPPS_4$ assemblies was then added onto the mica surface and kept still for 5 min to deposit these $TPPS_4$ assemblies. All the AFM images were taken in air using a MultiMode 8 AFM (Bruker, Germany) in the ScanAsyst mode with SNL-10 tips (Bruker, Germany) and analyzed by NanoScope Analysis software (Bruker, Germany).

Cryo-electron microscopy (Cryo-EM) For Cryo-EM characterization, aliquots of 3.5 μL of sample containing the $TPPS_4$ assemblies were applied to glow-discharged holey carbon grids (R1.2/1.3). After removing excess solution with filter paper, the specimens were vitrified by plunge freezing into a liquid ethane bath using the semi-automatic FEI Vitrobot Mark IV with blotting force of level 3, blotting time of 6 s, temperature of 22 ℃, and humidity of 100 %. Specimens were observed at −170 °C using a Model 626 cryo-

transfer specimen holder (Gatan, USA) with a Talos F200 transmission electron microscope (FEI, USA) under low-dose conditions at 200 kV. Images under electron dose of ~30 e Å^{-2} were directly acquired with a DE20 electron detector (Direct Electron, USA).

Trajectory of a single microsphere in inclined microchamber To track the motion of a single tracer particle in the inclined microchamber, we used a diluted particle suspension (0.001 %, w/v) of PS microspheres (1 μm in diameter, Phosphorex, USA) as the middle infusion. The observation was performed at optimized flow rate condition of 30–1–30 mL h^{-1}. Using a high speed CCD (Phantom v7.3, Vision Research Inc., USA) with a setup of 10^4 frames s^{-1}, the entire enter-and-exit event of a single particle was captured in 276 time-series frames (Supplementary Figure 13), suggesting a short residence time of ~ 27 ms.

Residence time within inclined microchannel. The residence times of particles in inclined microchambers were determined by the Lagrangian particle tracking of tracer particles using the CFD software Fluent 6.4 (Ansys Inc., USA). The steady flow field without tracer particles was first obtained by solving the Navier-Stokes equations. The tracer particles (1 μm in diameter) were then spiked from the right upstream of the microchambers and their motions were predicted by integrating the force balance of the particle based on a Lagrangian formulation:

$$\frac{d\mathbf{V}_\mathrm{p}}{dt} = \frac{18\eta}{\rho_\mathrm{p} a^2}\frac{C_\mathrm{D}\mathrm{Re}_\mathrm{s}}{24}\left(\mathbf{u}-\mathbf{V}_\mathrm{p}\right) + \frac{\mathbf{g}\left(\rho_\mathrm{p}-\rho\right)}{\rho_\mathrm{p}} + \frac{1}{2}\frac{\rho}{\rho_\mathrm{p}}\frac{d\left(\mathbf{u}-\mathbf{V}_\mathrm{p}\right)}{dt} \qquad (1)$$

where **u** is the flow velocity vector, $\mathbf{V}_\mathrm{p}$ the particle velocity vector, ρ_p the particle density, C_D the drag coefficient, Re_s the relative Reynolds number $\mathrm{Re}_\mathrm{s} = \rho a\left|\mathbf{u}-\mathbf{V}_\mathrm{p}\right|/\eta$, and **g** the

gravitational acceleration[1]. On the right hand of the force balance equation, the first term is the viscous drag force per unit particle mass, the second term is buoyant force that can be neglected for neutrally-buoyant particles, and the third term is the virtual mass force arising from the acceleration of the fluid around the particle. The residence time for a tracer particle was determined as the time interval between its entrance and exit of the microchamber. The average residence time for the spiked tracer particles (87 counts) was 36 ms with a maximal value of 100 ms.

Calculation of assembly time The self-assembly process was initiated spontaneously when the achiral BTAC ($TPPS_4$) building blocks were rotated within the chiral microvortices, and diffused into the DMF/H_2O (HCl/C_2mim^+) solutions. Therefore, we estimated the assembly time using the equation of the diffusion time $t = L^2/6D$. Here, L = 255 nm is the average dimension of primary nuclei and $D = kT/3\pi\eta a$ is the diffusion coefficient of molecules, where $k = 1.38\times10^{-23}\,\mathrm{J\,K^{-1}}$ is the Boltzmann constant, T = 298 K is temperature, $\eta = 10^{-3}$ Pa s is dynamic viscosity, and a is the size of molecule which is 30 Å for BTAC and 20 Å for $TPPS_4$. The estimated assembly time was 0.7 ms for BTAC and 0.5 ms for $TPPS_4$.

Calculation of the shear rate gradient The shear rate gradients in the x, y, and z directions were calculated as $\partial\gamma_x/\partial x$, $\partial\gamma_y/\partial y$, and $\partial\gamma_z/\partial z$, respectively. Here γ_x, γ_y, and γ_z are the shear rates accounting for the fluid rotation about the x, y, and z axes and expressed as $\partial u_z/\partial y - \partial u_y/\partial z$, $\partial u_x/\partial z - \partial u_z/\partial x$, and $\partial u_y/\partial x - \partial u_x/\partial y$, respectively.

Calculation of the shear force The hydrodynamic torque to twist the nuclei was originated from the shear force. For a $TPPS_4$/BTAC nucleus with a length of L

suspending in a flow field with a shear rate gradient Γ, the shear force exerting on the nucleus was estimated as $F_s = A\eta L\Gamma/2$, where A is the surface area of the nuclei determined as 1.5×10^{-13} m^2 for BTAC fiber and 1.1×10^{-14} m^2 for $TPPS_4$ tube. The Γ increased with off-wall distance and reached the maximum of 8×10^{10} m^{-1} s^{-1} at the half height of the microchamber, where the maximum F_s was estimated as 1.6 pN for BTAC nuclei and 0.1 pN for $TPPS_4$ nuclei.

Calculation of the Reynolds number The Reynolds number of the main channel was calculated as

$$\mathrm{Re} = \frac{U_{\mathrm{max}} D_{\mathrm{h}}}{\nu} = \frac{7\ \mathrm{m\ s^{-1}} \times 66.7\ \mu\mathrm{m}}{10^{-6}\ \mathrm{m^2 s^{-1}}} = 467$$

where U_{max} is the maximum channel velocity, D_{h} is the hydraulic diameter of the main channel that is expressed as $D_{\mathrm{h}} = 2WH/(W+H) = 66.7\ \mu\mathrm{m}$, and ν is the kinematic viscosity.

The Reynolds number of the microchamber was calculated as

$$\mathrm{Re} = \frac{U_{\mathrm{cham}} W_{\mathrm{cham}}}{\nu} = \frac{1.9\ \mathrm{m\ s^{-1}} \times 200\ \mu\mathrm{m}}{10^{-6}\ \mathrm{m^2 s^{-1}}} = 380$$

where U_{cham} is the average flow velocity at the junction of the microchamber and the main channel and W_{cham} is chamber width.

Supplementary References

1 Liu, C. *et al.* Field-free isolation of exosomes from extracellular vesicles by microfluidic viscoelastic flows. *ACS Nano* **11**, 6968-6976 (2017).
